# Global use of SGLT2 inhibitors and GLP-1 receptor agonists in type 2 diabetes. Results from DISCOVER

**DOI:** 10.1186/s12902-022-01026-2

**Published:** 2022-04-26

**Authors:** Suzanne V. Arnold, Fengming Tang, Andrew Cooper, Hungta Chen, Marilia B. Gomes, Wolfgang Rathmann, Iichiro Shimomura, Jiten Vora, Hirotaka Watada, Kamlesh Khunti, Mikhail Kosiborod

**Affiliations:** 1 Saint Luke’s Mid America Heart Institute, 4401 Wornall Rd, Kansas City, MO 64111 USA; 2 University of Missouri Kansas City, Kansas City, MO USA; 3 AstraZeneca, Cambridge, UK; 4 AstraZeneca, Gaithersburg, MD USA; 5 Rio de Janeiro State University, Rio de Janeiro, Brazil; 6 Institute for Biometrics and Epidemiology, German Diabetes Center, Leibniz Institute for Diabetes Research at Heinrich Heine University, Düsseldorf, Germany; 7 Graduate School of Medicine, Osaka University, Osaka, Japan; 8 University of Liverpool, Liverpool, UK; 9 Juntendo University, Tokyo, Japan; 10 University of Leicester, Leicester, UK; 11 The George Institute for Global Health and University of New South Wales, Sydney, Australia

**Keywords:** Diabetes, Cardiovascular disease, Quality of care

## Abstract

**Background:**

Despite strong evidence of benefit, uptake of newer glucose-lowering medications that reduce cardiovascular risk has been low. We sought to examine global trends and predictors of use of SGLT2i and GLP-1 RA in patients with type 2 diabetes.

**Methods:**

DISCOVER is a global, prospective, observational study of patients with diabetes enrolled from 2014–16 at initiation of second-line glucose-lowering therapy and followed for 3 years. We used hierarchical logistic regression to examine factors associated with use of either an SGLT2i or GLP-1 RA at last follow-up and to assess country-level variability.

**Results:**

Among 14,576 patients from 37 countries, 1579 (10.8%) were started on an SGLT2i (1275; 8.7%) or GLP-1 RA (318; 2.2%) at enrollment, increasing to 16.1% at end of follow-up, with large variability across countries (range 0–62.7%). Use was highest in patients treated by cardiologists (26.1%) versus primary care physicians (10.4%), endocrinologists (16.9%), and other specialists (22.0%; *p* < 0.001). Coronary artery disease (OR 1.29, 95% CI 1.08–1.54) was associated with greater use of SGLT2i or GLP-1 RA while peripheral artery disease (OR 0.73, 95% CI 0.54–1.00) and chronic kidney disease (OR 0.73, 95% CI 0.58–0.94) were associated with lower use (OR 0.73, 95% CI 0.54–1.00). The country-level median odds ratio was 3.48, indicating a very large amount of variability in the use of SGLT2i or GLP-1 RA independent of patient demographic and clinical factors.

**Conclusions:**

Global use of glucose-lowering medications with established cardiovascular benefits has increased over time but remains suboptimal, particularly in sub-groups most likely to benefit. Substantial country-level variability exists independent of patient factors, suggesting structural barriers may limit more widespread use of these medications.

## Background

Prior to 2015, neither a strategy of intensive glucose control nor individual glucose-lowering medications had been successful in reducing cardiovascular risk in patients with type 2 diabetes [1]. However, beginning with publication of the EMPA-REG OUTCOME [2] and Liraglutide Effect and Action in Diabetes: Evaluation of Cardiovascular Outcome Results (LEADER) trials [3], multiple trials and observational studies have shown cardiovascular risk reduction with the use of sodium-glucose co-transporter 2 inhibitors (SGLT2i) [4] and glucagonlike peptide-1 receptor agonists (GLP-1 RA) [5]. Despite this strong evidence of benefit, uptake has thus far been poor [6, 7], although notably these prior studies have been mostly cross-sectional studies from the US or Western European countries. We sought to more fully evaluate global trends in the use of these medications and to explore factors associated with differential use, including patient demographics, complications, physician specialty, and country.

## Methods

The DISCOVER study is a prospective, observational study of individuals with type 2 diabetes enrolled from 38 countries at initiation of second-line glucose-lowering medication [8, 9]. Consecutive eligible adults were enrolled between December 2014 and June 2016 and followed at 6 months and 1, 2, and 3 years. Exclusions included first-line therapy with an injectable agent or herbal/natural medicine alone, pregnancy, dialysis, or kidney transplant. For this specific analysis, we also excluded patients who were on an SGTL2i as first-line therapy (*n* = 115). Data from China were excluded (*n* = 1292) due to regulations on data privacy released during the study. In line with the observational nature of the study, data were recorded according to routine clinical practice. Comorbidities and events were not adjudicated and relied on the judgement of the local investigators. The study protocol was approved by the appropriate clinical research ethics committees in each participating country and by the institutional review board at each site. All participants provided written informed consent.

The primary outcome for this analysis was being treated with an SGLT2i or GLP-1 RA at the last visit for each patient. Use was compared across key comorbidities, country (grouped by geographic region and by gross national income per capita [10]), and physician specialty. Given the large cohort size, unadjusted comparisons were made using standardized differences, where differences of > 10% are considered clinically relevant. We then constructed a hierarchical logistic regression model to examine the association of patient factors with use of SGLT2i or GLP-1 RA at last study visit. Baseline patient factors included were age, sex, diabetes duration, smoking, body mass index, and systolic blood pressure. As diagnoses both at baseline and throughout follow-up could impact the prescription of one of these glucose-lowering medications, coronary artery disease (CAD; including myocardial infarction, coronary revascularization, angina), heart failure, cerebrovascular disease (including stroke, transient ischemic attack, carotid endarterectomy or stenting), peripheral artery disease (PAD; including diabetic foot, amputation), and chronic kidney disease (CKD) were included in the model as time-dependent covariates. Country was included as a random effect to account for patient clustering within countries, and country-level variability independent of patient factors was assessed with a median odds ratio. The median odds ratio estimates the differences in the odds of being on SGLT2i or GLP-1 RA between two patients with identical risk factors from two randomly selected countries. Median odds ratios are always ≥ 1, with higher values indicating increased country-level variability in SGLT2i or GLP-1 RA use independent of patient factors. All analyses were conducted using SAS version 9.4 (SAS Institute, Cary, North Carolina), with statistical significance determined by *p* < 0.05.

## Results

Among 14,576 patients with diabetes from 37 countries, mean age was 57.5 ± 12.0 years, 6718 (46.1%) were women, mean diabetes duration was 5.7 ± 5.3 years, and mean HbA1c was 67 ± 7 mmol/mol (8.3 ± 1.7%). At enrollment, 1579 patients (10.8%) were started on an SGLT2i or GLP-1 RA as second-line glucose-lowering treatment (1261 [8.7%] SGLT2i only, 304 [2.1%] GLP-1 RA only, 14 [0.1%] both), which increased over the 3 years of follow-up, such that at last study visit, 2348 patients (16.1%) were on an SGLT2i or GLP-1 RA (1870 [12.8%] SGLT2i only, 376 [2.6%] GLP-1 RA only, 102 [0.7%] both). Patient characteristics of those on versus not on an SGLT2i or GLP-1 RA are shown in Table 1.

**Table 1:** Characteristics of patients treated versus not treated with SGLT2i or GLP-1 RA at last follow-up

	**SGLT2i or GLP-1 RA**		**Standardized Difference (%)**^**a**^
	**Yes** ***n* = 2348**	**No** ***n* = 12,228**	
**Patient factors at enrollment**			
Age (years)	56.1 ± 11.5	57.7 ± 12.1	13.8
Female	42.4%	46.8%	8.8
Education level			26.2
Primary or none	10.1%	20.0%	
Secondary	51.6%	49.1%	
University	37.3%	30.9%	
Current smoker	18.0%	12.5%	21.5
Body mass index (kg/m^2^)	31.8 ± 6.5	28.9 ± 5.8	47.0
Duration of diabetes	5.6 ± 5.0	5.8 ± 5.3	3.9
HbA1c (%)	8.3 ± 1.6	8.3 ± 1.7	2.6
Creatinine (mg/dL)	0.9 ± 1.0	1.0 ± 1.0	5.1
Specialty of Main Investigator			28.3
Primary care	20.2%	31.4%	
Endocrinology	74.0%	65.5%	
Cardiology	4.7%	2.4%	
Other	1.1%	0.7%	
Region			60.3
Africa	0.6%	6.5%	
Americas	18.1%	12.8%	
Europe	34.1%	21.8%	
Middle East	11.7%	15.5%	
Southeast Asia	10.3%	25.4%	
Western Pacific	25.2%	17.9%	
Comorbidities at last follow-up			
Coronary artery disease	14.8%	10.1%	14.4
Cerebrovascular disease	2.9%	2.9%	0.2
Heart failure	7.5%	4.3%	13.2
Peripheral artery disease	3.4%	3.3%	0.6
Chronic kidney disease	6.1%	5.1%	4.6

^a^ > 10% difference is considered clinically relevant

### Use by patient comorbidity, country, and physician specialty

**Fig.1:**
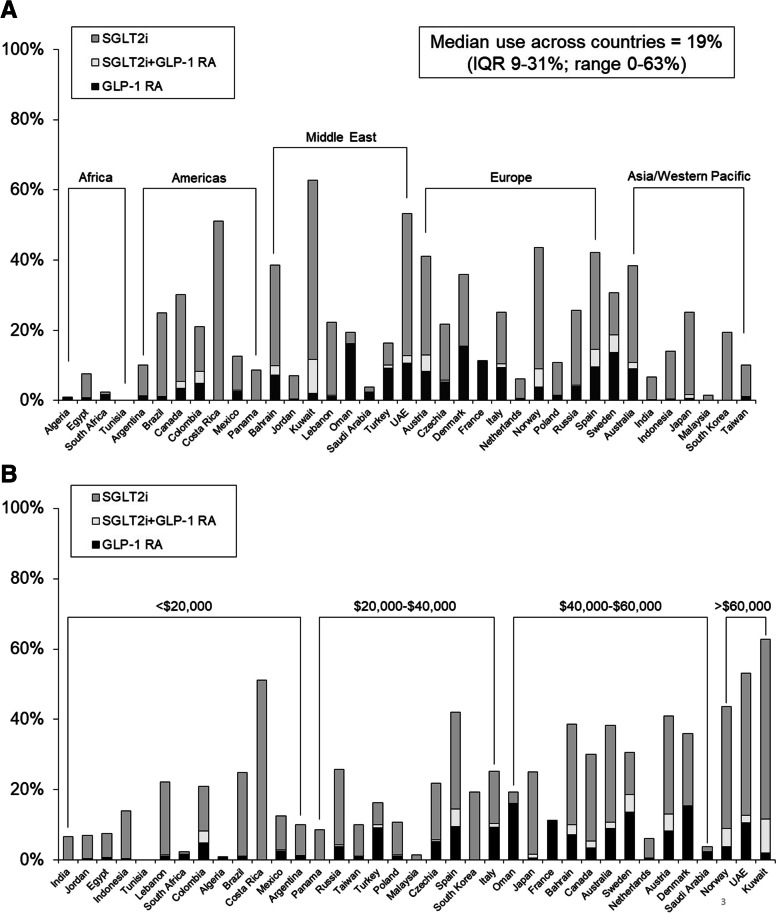
Use of SGLT2 inhibitors and GLP-1 receptor agonists by country. **A** Grouped by global region; **B** Ordered by gross national income per capita

Patients with (vs. without) CAD, heart failure, and CKD were more likely to be on SGLT2i or GLP-1 RA (CAD: 20.0% vs. 13.8%; heart failure: 22.5% vs. 14.1%; CKD: 17.1% vs. 14.4%; all *p* < 0.001), whereas use was similar in those with vs. without cerebrovascular disease (14.7% vs. 14.5%, *p* = 0.18) and PAD (14.9% vs. 14.5%, *p* = 0.11). The median use of either SGLT2i or GLP-1 RA at end of follow-up across the 37 countries was 19.4% (IQR 8.7–30.6%; range 0–62.7%). Countries in Africa and Asia had notably low rates of use (Fig. 1A), and there was a trend toward higher use in countries with greater economic resources (Fig. 1B). Finally, use of SGLT2i and GLP-1 RA in patients treated by primary care physicians (*n* = 4105) was 10.4% [SGLT2i 7.7%, GLP-1 RA 3.2%]; endocrinologists (*n* = 9234): 16.9% [SGLT2i 14.7%, GLP-1 RA 2.8%]; cardiologists (*n* = 380): 26.1% [SGLT2i 25.0%, GLP-1 RA 1.1%]; and other specialists (*n* = 109): 22.0% [SGLT2i 13.8%, GLP-1 RA 9.2%] (*p* < 0.001).

**Fig. 2:**
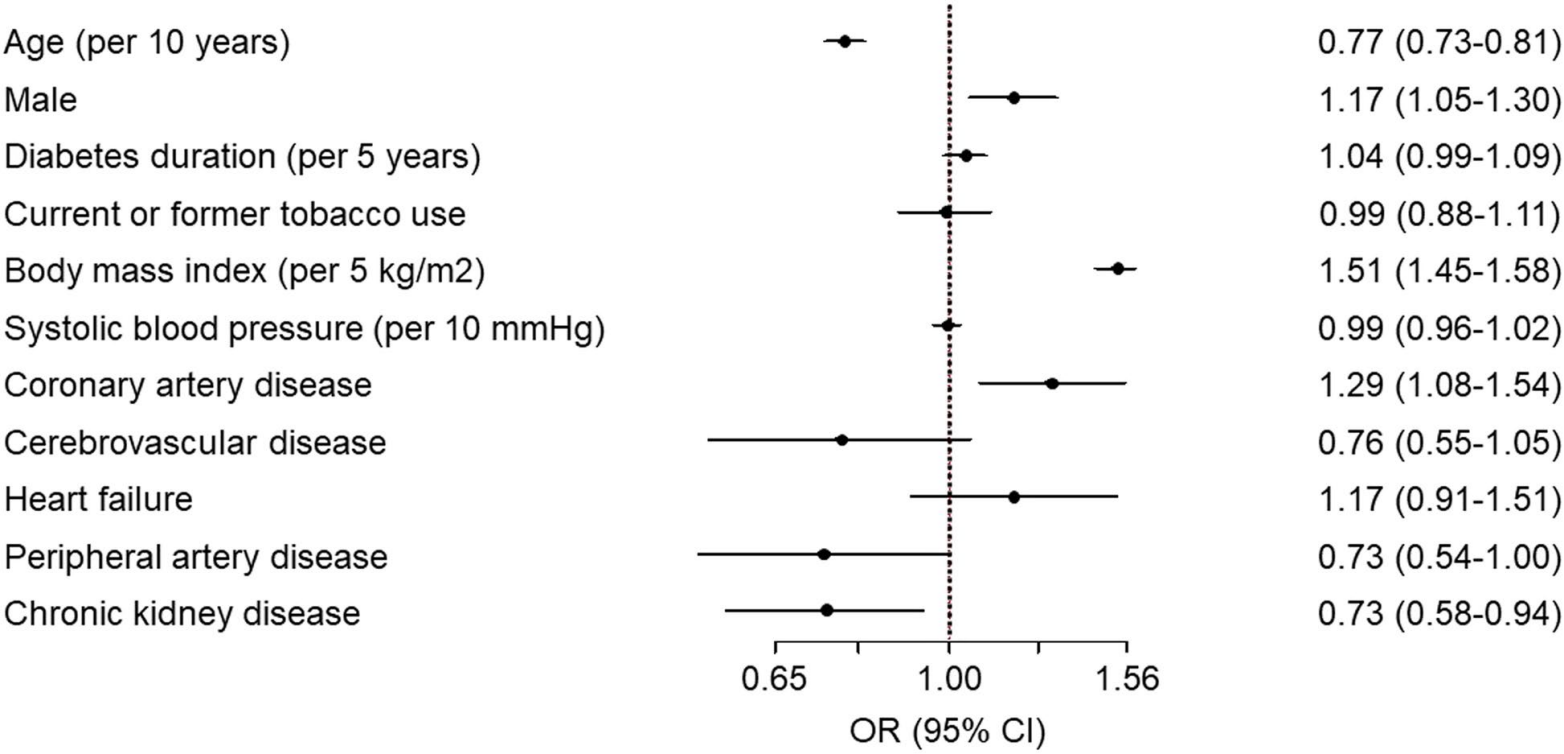
Use of SGLT2 inhibitors or GLP-1 receptor agonists by patient factors according to the hierarchical logistic regression model

In the hierarchical logistic regression model, younger age (OR 0.77 per 10 year increase, 95% CI 0.73–0.81), male sex (OR 1.17, 95% CI 1.05–1.30), and higher body mass index (OR 1.51 per 5 kg/m^2^, 95% CI 1.45–1.58) were associated with a greater use of SGLT2i or GLP-1 RA (Fig. 2). In terms of comorbidities/cardiovascular events (both prior to enrollment and during follow-up), CAD (OR 1.29, 95% CI 1.08–1.54) was associated with greater use of SGLT2i or GLP-1 RA while PAD and CKD were associated with lower use (PAD: OR 0.73, 95% CI 0.54–1.00; CKD: OR 0.73, 95% CI 0.58–0.94). The country-level median odds ratio was 3.48, indicating a very large amount of variability in the use of SGLT2i or GLP-1 RA independent of patient demographic and clinical factors.

## Discussion

In a large, multinational, prospective cohort study of patients with type 2 diabetes enrolled at the time of initiation of second-line glucose-lowering medication, we found that use of glucose-lowering medications shown to have cardiovascular risk reduction has increased over time but remains suboptimal. The majority of increase was observed in the use of SGLT2i, with particularly high use among cardiologists. Although patients with CAD, heart failure, and CKD were more likely to be on these medications compared with patients without these conditions, after accounting for patient factors and concomitant comorbidities, only CAD was associated with a greater use of either SGLT2i or GLP-1 RA while PAD and CKD were associated with lower use. Finally, we saw a substantial degree of variability in the use of these medications across countries—both unadjusted and after accounting for patient factors and comorbidities—suggesting that structural barriers likely continue to limit broader use of these medications.

### Prior studies

Prior studies have shown SGLT2i or GLP-1 RA uptake to be suboptimal, despite a number of trials and observational studies providing evidence of cardiovascular benefit and several position papers and guideline statements encouraging broader use [1, 11]. A recent study of ~ 10,000 patients from 13 countries with diabetes found 22% of patients were on an SGLT2i or GLP-1 RA (15% SGLT2i, 9% GLP-1 RA), with no differences according to the presence or absence of CAD or of cardiovascular disease [7]. We found use to be lower in our study, which likely represents differences in the enrolling countries, as countries in DISCOVER had a broader spectrum of socioeconomic status (e.g., Africa and Central America). The large country-level variability in the use of SGLT2i or GLP-1 RA in DISCOVER illustrates the importance of healthcare policy and access in the use of these medications and exposes the need to target not only individual physicians but structural issues within the healthcare system that could allow physicians to treat patients with the optimal medications. Notably, we found that patients in DISCOVER who had CAD were more likely to be treated with SGLT2i or GLP-1 RA. While more education continues to be needed about the potential benefit of these medications across the spectrum of cardiovascular and kidney disease, it is encouraging to see at least some targeted use of these medication in patients who are most likely to benefit.

### Limitations

Although DISCOVER included many lower-income countries that have rarely been studied, the cohort remains under-representative of very poor countries in addition to patients within these countries who did not have access to medical care, both of which would lead to lower use of SGLT2i or GLP-1 RA. In addition, we did not have data on individual access to medications (e.g., medication coverage, socioeconomic status) and could only observe country-level effects.

## Conclusion

In a large, multinational, prospective cohort study of patients with type 2 diabetes, use of glucose-lowering medications with cardiovascular risk reduction has increased over time (particularly SGLT2i) but remains suboptimal. While we observed some targeted use of SGLT2i or GLP-1 RAs in patients with CAD, other comorbidities (e.g., heart failure, chronic kidney disease) were not associated with increased use despite the known benefits in these clinical settings. Substantial country-level variability exists—both unadjusted and after accounting for patient factors and comorbidities—suggesting that structural barriers likely continue to limit broader use of these medications.

## Data Availability

The data that support the findings of this study are available from AstraZeneca. Restrictions apply to the availability of these data, which were used under license for this study. Data are available from the authors with the permission of AstraZeneca.

## Abbreviations

SGLT2i: Sodium-glucose co-transporter 2 inhibitors
GLP-1 RA: Glucagonlike peptide-1 receptor agonists
CAD: Coronary artery disease
PAD: Peripheral artery disease
CKD: Chronic kidney disease

## References

[1] Arnold SV, Bhatt DL, Barsness GW, Beatty AL, Deedwania PC, Inzucchi SE, Kosiborod M, Leiter LA, Lipska KJ, Newman JD (2020). Clinical management of stable coronary artery disease in patients with Type 2 Diabetes Mellitus: a scientific statement from the American heart association. Circulation.

[2] Zinman B, Wanner C, Lachin JM, Fitchett D, Bluhmki E, Hantel S, Mattheus M, Devins T, Johansen OE, Woerle HJ (2015). Empagliflozin, cardiovascular outcomes, and mortality in Type 2 Diabetes. N Engl J Med.

[3] Marso SP, Daniels GH, Brown-Frandsen K, Kristensen P, Mann JF, Nauck MA, Nissen SE, Pocock S, Poulter NR, Ravn LS (2016). Liraglutide and cardiovascular outcomes in Type 2 Diabetes. N Engl J Med.

[4] McGuire DK, Shih WJ, Cosentino F, Charbonnel B, Cherney DZI, Dagogo-Jack S, Pratley R, Greenberg M, Wang S, Huyck S (2021). Association of sglt2 inhibitors with cardiovascular and Kidney outcomes in patients with Type 2 Diabetes: a meta-analysis. JAMA Cardiol.

[5] Kristensen SL, Rorth R, Jhund PS, Docherty KF, Sattar N, Preiss D, Kober L, Petrie MC, McMurray JJV (2019). Cardiovascular, mortality, and kidney outcomes with GLP-1 receptor agonists in patients with type 2 diabetes: a systematic review and meta-analysis of cardiovascular outcome trials. Lancet Diabetes Endocrinol.

[6] Arnold SV, Inzucchi SE, Tang F, McGuire DK, Mehta SN, Maddox TM, Goyal A, Sperling LS, Einhorn D, Wong ND (2017). Real-world use and modeled impact of glucose-lowering therapies evaluated in recent cardiovascular outcomes trials: an NCDR(R) Research to Practice project. Eur J Prev Cardiol.

[7] Vencio S, Alguwaihes A, Arenas JL, Bayram F, Darmon P, Dieuzeide G, Hettiarachchige N, Hong T, Kaltoft MS, Lengyel C (2020). Contemporary use of diabetes medications with a cardiovascular indication in adults with type 2 diabetes: a secondary analysis of the multinational CAPTURE study. 56th EASD Annual Meeting of the European Association for the Study of Diabetes. Diabetologia.

[8] Khunti K, Ji L, Medina J, Surmont F, Kosiborod M (2019). Type 2 diabetes treatment and outcomes worldwide: A short review of the DISCOVER study programme. Diabetes Obes Metab.

[9] Ji L, Bonnet F, Charbonnel B, Gomes MB, Kosiborod M, Khunti K, Nicolucci A, Pocock S, Rathmann W, Shestakova MV (2017). Towards an improved global understanding of treatment and outcomes in people with type 2 diabetes: Rationale and methods of the DISCOVER observational study program. J Diabetes Complications.

[10] World Bank Country Categorization by GNI/capita. http://databank.worldbank.org/data/download/site-content/OGHIST.xls. Accessed 1 Oct 2019.

[11] Buse JB, Wexler DJ, Tsapas A, Rossing P, Mingrone G, Mathieu C, D'Alessio DA, Davies MJ (2020). 2019 Update to: Management of Hyperglycemia in Type 2 Diabetes, 2018. a consensus report by the American Diabetes Association (ADA) and the European association for the study of Diabetes (EASD). Diabetes Care.

